# Anatomy learning profiles in relation to student motivation and academic success: A multi‐center cross‐sectional study

**DOI:** 10.1002/ase.70065

**Published:** 2025-06-27

**Authors:** Birte Barbian, Thomas Shiozawa, Morris Gellisch, Irene Brunk, Katharina Langer‐Fischer, Nicole Wagner, Sina Benker, Michelle Bellstedt, Nils Otto, Dogus Darici

**Affiliations:** ^1^ Institute of Anatomy and Molecular Neurobiology University of Münster Münster Germany; ^2^ Department of Anatomy, Institute of Clinical Anatomy and Cell Analysis, Faculty of Medicine Eberhard Karls University of Tuebingen Tuebingen Germany; ^3^ Department of Anatomy and Molecular Embryology, Institute of Anatomy, Medical Faculty Ruhr University Bochum Bochum Germany; ^4^ Center for Medical Education Ruhr‐University Bochum Bochum Germany; ^5^ Institute for Integrative Neuroanatomy Charité‐Universitätsmedizin Berlin Berlin Germany; ^6^ Institute of Molecular and Cellular Anatomy Ulm University Ulm Germany; ^7^ Institute of Institute for Anatomy and Cell Biology University of Würzburg Würzburg Germany

**Keywords:** anatomy education, anatomy learning profiles, latent profile analysis, multicenter study

## Abstract

Learning strategies encompass the diverse approaches students employ to master challenging subjects like anatomy. However, research examining these strategies in isolation remains inconclusive. Recent evidence suggests a shift toward a person‐centered paradigm that considers how student subgroups combine multiple learning strategies. This study advances this paradigm and identifies Anatomy Learning Profiles (ALPs) among 567 medical and dental students across six medical faculties by analyzing 13 self‐reported cognitive, metacognitive, and resource management strategies using the LIST‐K questionnaire. A latent profile analysis confirmed the existence of four ALPs among the study population: Active (26%), Collaborative (20%), Structured (39%), and Passive (15%). Each profile represented a unique combination of 13 learning strategies and was significantly shaped by individual and institutional factors. For example, students further along in their training were more likely to exhibit traits of the Collaborative ALP and less of the Structured ALP. Female and younger students predominantly adopted Structured profiles, while older students favored the Active profile. Moreover, students in modular curricula showed higher rates of Collaborative profiles, while traditional curricula fostered Structured profile membership. Importantly, Active and Collaborative ALP students outperformed the Structured and Passive ALPs, and Active ALP students demonstrated the highest motivation levels. Yet, despite lower motivation levels, Collaborative ALP students achieved comparable academic success, indicating that studying with peers may compensate for lower individual motivation. These findings contribute to understanding students' learning strategies and may provide a holistic basis for educational interventions in anatomy education that align with students' combined learning behaviors.

## INTRODUCTION

Students' learning processes largely occur outside the classroom, and their study practices are often elusive to anatomy educators. Due to anatomy's information‐rich nature and the truncation of curricular teaching time (e.g., Singh et al.^1^), future medical and dental professionals face mounting challenges when self‐organizing their learning processes. To handle this, each individual might employ different learning strategies to study anatomy, such as extensive rehearsal and effort regulation, elaboration and critical thinking, or spending time learning with peers. Thirteen of such goal‐oriented learning behaviors have been identified in commonly acknowledged models and subsumed under four overarching dimensions: cognitive and metacognitive learning strategies and management of internal and external resources.^2^, ^3^ Ever since, anatomy educators have worked to understand which learning strategies are used within their educational context and have attempted to link these strategies to learning outcomes. Classifying the 13 learning strategies according to their effectiveness for learning anatomy may be practically useful for making recommendations to students (e.g., Pandey and Zimitat^4^; Wang and McWatt^5^).

V*ariable‐centered studies* that focus on single variables instead of combinations of predictors have demonstrated inconsistent associations between isolated learning strategies and academic success. For example, while Pizzimenti and Axelson^6^ found elaboration and critical thinking to correlate moderately with anatomy course performance, Zilundu et al.^7^ found no significant correlations of either of these cognitive strategies. Otto et al.^8^ addressed this limitation by introducing a *person‐centered approach* in anatomy education research, examining how students combine multiple learning strategies rather than analyzing isolated strategies or individual variables. Using a person‐centered analysis, they clustered medical students into four distinct Anatomy Learning Profiles (*Active, Collaborative, Structured, and Passive ALPs*) based on students' combined usage of learning strategies. Using a similar approach, Odontides et al.^9^ identified three clusters of ALPs (*Meta‐strategy‐learner, Cognitive‐strategy‐learner, and Mixed‐strategy‐learner*). This methodological approach represents a significant shift from traditional variable‐centered research, which typically examines individual learning strategies as single variables. By analyzing how students integrate multiple learning strategies simultaneously, there may be an opportunity to develop a more comprehensive understanding of how students study anatomy.

The two currently available person‐centered studies in anatomy education, while promising, faced several limitations. Firstly, the most notable constraint was their exclusive focus on gross anatomy, creating a substantial knowledge gap regarding learning profiles in other anatomical subdisciplines such as histology, embryology, and neuroanatomy. This limitation was particularly significant given Kim et al.'^10^ findings that specific disciplinary demands can substantially influence how medical students implement their learning strategies. Furthermore, research by O'Connell et al.^11^ and Quintero et al.^12^ has shown that different curricular approaches in medical education may impact both student knowledge acquisition and subjective attitudes toward learning. These findings from the broader medical education context suggest that both the structure of anatomy curricula and disciplinary context may also play a role in shaping students' learning behaviors.

Secondly, Otto et al.'s^8^ reliance on self‐reported grades warrants consideration, as such data may not fully reflect true academic performance due to reporting biases and recall inaccuracies. In addition, the external validity of Odontides et al.'s^9^ findings was constrained by their single‐institution study design. While their research provides valuable insights, the generalizability of their results to other educational contexts remains uncertain, particularly given the potential influence of institutional factors such as curriculum design, teaching methods, and student demographics on learning profiles.

The current study aimed to address the limitations of existing person‐centered studies in anatomical sciences education and expand the understanding of ALPs by analyzing motivational variables that may influence ALP membership. While learning strategies represent the behavior patterns students employ, these patterns are driven by motivational constructs that influence how and why students engage with academic content. Based on the established general education and medical education literature, four key motivational constructs with particular relevance to anatomy learning were selected: Self‐efficacy,^13^, ^14^ competitiveness,^15^, ^16^, ^17^ work mastery,^18^ and mastery goal orientation.^19^, ^20^ These motivational constructs may serve as underlying drivers that help explain why students adopt certain combinations of learning strategies, thus forming distinct ALPs. Furthermore, by analyzing objective anatomy examination scores across multiple institutions, including both medical and dental students studying various anatomical subdisciplines, the current study aims to provide a more comprehensive understanding of how learning profiles relate to academic outcomes in anatomy education. The paradigm shift in ALP research promises to transform not only how anatomy learning is researched but also how students can be aided to improve their learning processes. This work may inform how students should combine and adapt their learning strategies across different anatomical contexts, providing insight into the dynamic nature of learning strategy development. Integrating motivational factors and objective performance measures across multiple institutions creates a framework for understanding how learning profiles evolve throughout anatomical education, serving as a feedback mechanism for educators and potentially leading to more tailored and effective educational interventions.

### Learning styles versus learning profiles

This methodological transition from variable‐centered to person‐centered approaches reflects a broader conceptual shift from learning styles to learning profiles in educational research. While learning styles research has traditionally categorized students based on presumed stable preferences (such as visual, auditory, or kinesthetic),^21^ learning profiles represent a more dynamic, multidimensional conceptualization that captures how students strategically combine various learning strategies. Unlike the trait‐based conception of learning styles, learning profiles acknowledge that individuals adaptively integrate multiple strategies in response to specific educational contexts and demands. This distinction is particularly relevant in challenging disciplines like anatomy, where students may employ diverse combinations of strategies to master complex material rather than relying on singular stylistic preferences. In addition, meta‐analyses have questioned the empirical basis for matching teaching methods to presumed learning styles, with studies like Husmann and O'Loughlin^21^ finding no correlation between VARK learning styles and academic performance in anatomy. For a detailed description of the theoretical foundations and measurement models, see Supporting Information Appendix S1.

### Study purpose

Four research questions (RQs) guided this work:

RQ1—How reproducible are the four ALPs identified by Otto et al.^8^ within the context of a multicenter sample inclusive of anatomical subdisciplines and medical and dental students?

RQ2—To what extent do ALPs differ, if at all, across institutions, semesters, disciplines, students' prior work experiences, age categories, and gender?

RQ3—How well do the four ALPs relate to academic success (i.e., course performance)?

RQ4—How well do the four ALPs relate to motivational factors, including self‐efficacy, competitiveness, work mastery, and mastery goal orientation?

By integrating motivational constructs with the existing framework of learning strategies, this study aims to develop a more comprehensive understanding of how motivational factors and learning behaviors vary across different Anatomy Learning Profiles.

## METHODS

### Study population and participant recruitment

This cross‐sectional multicenter study was conducted at the University of Münster, Germany, in accordance with the Declaration of Helsinki. The study protocol was reviewed by the university's ethics committee and deemed not to require formal medical ethics approval (reference: 2023–073‐f‐N). Informed consent was obtained from all participants. The “journal recommended guidelines for survey‐based research”^22^ provided a framework for the methodological approach and were followed throughout our survey design, implementation, and reporting.

Medical and dental students in their first to fourth semesters from six German universities (Münster, Bochum, Charité Berlin, Würzburg, Tübingen, and Ulm) were invited via email or during regular anatomy courses, including gross anatomy, histology, and embryology. The participating universities follow varying curricular structures in anatomy education. Five of the universities (Münster, Tübingen, Bochum, Würzburg, and Ulm) use traditional subject‐based curricula where the anatomical sciences are taught as distinct subdisciplines (i.e., microscopic anatomy, macroscopic anatomy, and neuroanatomy), with differences in the sequencing and distribution of courses across semesters. For example, Münster offers macroscopic anatomy mainly in the second semester, while Ulm's program expands the material into three semesters. In contrast, Charité Berlin follows a modular curriculum, where anatomy is integrated into organ system modules spread across multiple semesters. For further curricular details, refer to the respective institutional websites.^23^, ^24^, ^25^, ^26^, ^27^, ^28^

Although this study employs a cross‐sectional design, which limits causal interpretations of profile development over time, semester‐level analysis provides information on the potential temporal evolution of ALPs during the early years of medical education. While this approach may offer some insights into how profile distributions may shift across different stages of anatomy learning, only a longitudinal study design can track individual profile changes definitively. The employed cross‐sectional design enabled simultaneous data collection across six different institutions with diverse curricular approaches, providing a multi‐institutional perspective. The specific timing at the end of the summer semester allowed for capturing students' learning strategies after they had developed established approaches to their anatomy coursework, providing data on settled rather than transitional learning behaviors. Additionally, this approach aligns with the methodological framework established by Otto et al.,^8^ facilitating direct comparisons with their findings.

### Measurements

The 69 item survey (Table 1) consisted of Likert‐scale items and questions about age and sociodemographics (i.e., gender, study subject, semester, prior vocational training). Learning strategies were assessed using the LIST‐K,^2^ while the self‐efficacy scale was adopted from the General Self‐Efficacy Scale.^29^ The competitiveness and work mastery scales were retrieved from Elliot and McGregor,^30^ and the mastery goal orientation scale was retrieved from the “PISA 2018 Scale Handbook and Documentation of Survey of Instruments”.^31^ The survey did not utilize any branching or skipping logic, though participants could navigate backwards through the questions. There was no time limit for survey completion. The survey was conducted between July and August 2024, which corresponds to the end of the summer semester in Germany. Study participation was truly voluntary (i.e., participants had no known ties to the survey distributors), and one reminder was sent. No incentives were offered.

**TABLE 1:** Characteristics of survey instruments.

Instruments	Number of items	Response‐range with labeled anchors	Sample item	*α*_mean_	Source
Learning strategies (List‐K)	39 items (across 13 first order latent factors)	1 = “very rarely” to 5 = “very often”	“What I learn, I also examine critically.”	0.838	Klingsieck^2^
Self‐efficacy	10 items	1 = “do not agree” to 4 = “fully agree”	“When a problem arises, I can overcome it on my own.”	0.865	Schwarzer and Jerusalem^29^
Competitiveness	3 items	1 = “do not agree” to 4 = “fully agree”	“I enjoy working in situations where I am in competition with others.”	0.793	Elliot and McGregor^30^
Work mastery	4 items	1 = “do not agree” to 4 = “fully agree”	“When I start a task, I do not give up until I have completed it.”	0.638	Elliot and McGregor^30^
Mastery goal orientation	3 items	1 = “does not apply” to 5 = “fully applies to me”	“My goal is to completely master the material that was covered.”	0.619	Mang et al.^31^

Abbreviation: *α*, Cronbach's alpha coefficient.

### Academic success in anatomy

Participants were asked to report on their anatomy examination score numerically: “If applicable, please provide your score from the last anatomy examination”, and “In your last exam, what was the highest score achievable?”. Afterward, the score was normalized to rescale the input values to a specific range (0%–100%).

### Data analysis

Missing values (around two percent of the whole dataset) were replaced using multiple imputation as per the methods described by Otto et al.^8^ Statistical analyses were performed using R^32^ and IBM SPSS Statistics v. 29 (IBM Corp., Armonk, NY). The figures were produced using IBM SPSS Statistics v. 29 (IBM Corp., Armonk, NY) and GraphPad PRISM 10.3.1 (GraphPad Software, Inc., San Diego, CA). Statistical testing was performed two‐sided at a significance level of *α* = 0.05. Latent Profile Analysis (LPA), using the OpenSource R package tidyLPA,^33^ was used to cluster the students based on their use of thirteen learning strategies, following the methodological approach by Otto et al.^8^ To determine the optimal number of profiles, multiple models (two to five profiles) were compared using established fit indices (Bayesian Information Criterion [BIC], sample‐size adjusted BIC [SABIC], and Akaike Information Criterion [AIC]) with lower values indicating better model fit.^34^ Classification quality was evaluated using entropy, with values greater than 0.60 considered acceptable,^34^ and posterior classification probabilities above 0.80 supporting distinct profile separation. The Bootstrapped Likelihood Ratio Test (BLRT) was applied to compare neighboring models, favoring the more parsimonious solution when *p* > 0.05. In addition to statistical fit, model selection was informed by theoretical interpretability and the requirement for substantial, non‐redundant profiles with realistic subgroup sizes. One‐way ANOVAs with Bonferroni‐corrected post‐hoc tests were applied to compare the mean values between the ALPs.

## RESULTS

### Participant characteristics and descriptive statistics

After exclusions, a total of 567 participants were used in the final analyses, representing around 20% of the students who were invited to participate (Table 2). Ten students did not provide information about their current semester, though they completed all other aspects of the survey and were fully included in the analyses. Extreme values (defined as above or under 3 standard deviations) in any scales were retained as they are expected in larger samples. Only two percent of the responses contained missing values, which were imputed. Mean ages were comparable across institutions, ranging from 21.1 (Tübingen, Ulm) to 22.7 (Berlin) years of age. The overall sample showed a predominance of female students (females = 410, males = 152, diverse = 5), as is typical for a German medical student population. Students were predominantly in early semesters, with means ranging from 2.16 (*σ* = 0.52) in Ulm to 3.06 (*σ* = 1.16) in Berlin. Medical students constituted the majority (*n* = 511) and dental students the minority (*n* = 46). Moreover, 114 students (20.1%) reported having completed vocational training prior to medical school, with proportions varying across institutions from 12.9% (Ulm) to 26.0% (Würzburg). The survey instruments demonstrated appropriate reliability with Cronbach's alpha coefficients ranging from 0.619 to 0.865 as detailed in Table 1.

**TABLE 2:** Sociodemographic characteristics of medical and dental students across six German institutions.

Characteristics	Study location					
	Münster (*n* = 170)	Berlin (*n* = 96)	Bochum (*n* = 55)	Tübingen (*n* = 138^a^)	Ulm (*n* = 31)	Würzburg (*n* = 77^a^)
Age (years)	21.15 (*σ* = 2.38)	22.65 (*σ* = 3.43)	21.63 (*σ* = 3.03)	21.06 (*σ* = 2.58)	21.06 (*σ* = 3.31)	21.76 (*σ* = 3.16)
Gender	f = 135 m = 34 d = 1	f = 70 m = 25 d = 1	f = 38 m = 17 d = 0	f = 93 m = 43 d = 2	f = 19 m = 12 d = 0	f = 55 m = 21 d = 1
Vocational training	yes = 29 no = 141	yes = 23 no = 73	yes = 13 no = 42	yes = 25 no = 113	yes = 4 no = 27	yes = 20 no = 57
Semester (mean)	2.64 (*σ* = 0.70)	3.06 (*σ* = 1.16)	3.02 (*σ* = 0.99)	2.49 (*σ* = 1.09)	2.16 (*σ* = 0.52)	3.05 (*σ* = 0.83)
Dental students	32	0	0	6^a^	1	14^a^
Medical students	138	96	55	130^a^	30	62^a^

Abbreviations: d, diverse (i.e., non‐binary); f, female; m, male; *σ*, standard deviation.

^a^ Numbers do not always add up due to missing values.

### Latent profile solution (RQ1)

**FIGURE 1:**
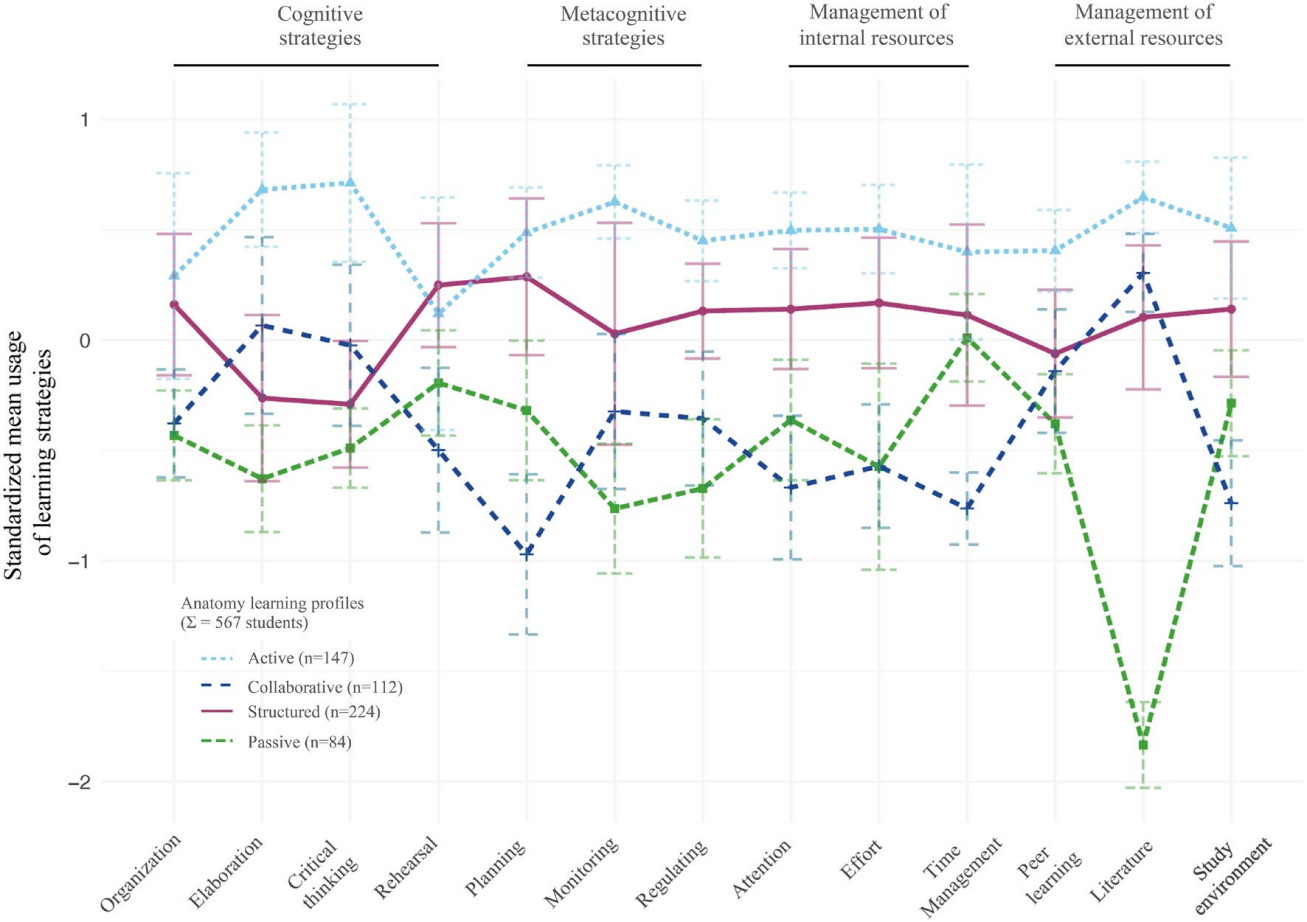
Identification of four distinct Anatomy Learning Profiles (ALPs) using a person‐centered approach. Results of the Latent Profile Analysis are shown based on students' 13 learning strategy usage across cognitive, metacognitive, and resource management domains of the LIST‐K questionnaire. Standardized *y*‐axis scores indicate the frequency of learning strategy usage. A value of 0 indicates average usage. The lines indicate profile membership. The color coding is as follows: Light blue (Active ALP), dark blue (Collaborative ALP), burgundy (Structured ALP), and green (Passive ALP).

Based on the 13 learning strategies of the LIST‐K questionnaire, latent profile analysis (LPA) confirmed the existence of the four distinct ALPs (Figures 1 and S1). The selection of the 4‐profile solution is supported by criteria defined in.^8^ The solution represents an optimal balance between model fit and parsimony. For example, while the BIC showed a negligible increase from the 3‐ to 4‐profile solution (just 2 points, from 20,499 to 20,501), the SABIC demonstrated a substantial improvement in fit (decreasing from 20,327 to 20,286) when accounting for sample size. The AIC also showed meaningful improvement with the 4‐profile solution (20,264 to 20,206), suggesting enhanced model fit.

The classification quality remains robust with the 4‐profile solution. Although the entropy value slightly decreases from the 3‐profile solution (0.779 to 0.702), it maintains an acceptable level above 0.70. Notably, the minimal entropy change from 4 to 5 profiles (0.702–0.711) suggests limited benefit in adding a fifth profile. The 4‐profile solution also yielded practically meaningful group sizes, with the smallest profile comprising 14.8% of cases and the largest containing 39.5%. This distribution is more balanced than the 3‐profile solution, where the largest group encompasses 56.6% of cases, and more robust than the 5‐profile solution, where the smallest group drops to 13.2%.

From a substantive perspective, the 4‐profile solution strikes an optimal balance between differentiation and interpretability. It provides sufficient complexity to capture meaningful patterns in the data while avoiding overly fine distinctions that might lack theoretical significance. The profile sizes are large enough to be practically meaningful for subsequent analyses and interpretations, yet distinct enough to represent meaningful subgroups in the population. This combination of statistical evidence and theoretical utility makes the 4‐profile solution the most appropriate choice for this analysis.

### Learning strategy use of the anatomy learning profiles

Each of the four ALPs was characterized by unique patterns in their use of the 13 Learning Strategies (Figure 1). The names of the profiles were kept similar to those used by Otto et al.^8^ to ensure comparability. *Active ALP students* (*n* = 147, dotted light blue line, Figure 1) showed consistently above‐average usage across most strategies, with notable peaks in elaboration and critical thinking within the cognitive domain. This profile maintains positive values across metacognitive strategies, with a slight dip in rehearsal, but generally remains above the mean. *Collaborative ALP* students (*n* = 112, dashed blue line, Figure 1) displayed variable usage patterns, alternating between positive and negative standardized scores. While showing peaks in the use of literature and elaboration, they demonstrated lower scores in metacognitive strategies, particularly in planning and time management. *Structured ALP* students (*n* = 224, solid burgundy line, Figure 1) exhibited the most consistent pattern, with scores clustering around the mean. They showed slight elevations in rehearsal, planning, and organization strategies, slightly below‐average use of elaboration and critical thinking, and maintained relatively stable scores across metacognitive strategies and management of resources, without extreme highs or lows. *Passive ALP students* (*n* = 84, dotted green line, Figure 1) showed below‐average usage of most strategies, with their lowest in literature usage. However, they showed distinctive peaks in time management, peer learning, and rehearsal. This profile displays the most fluctuations among all groups, particularly in the management of external resources.

### Characteristics of the anatomy learning profiles (RQ2)

#### Geographical distribution

**FIGURE 2:**
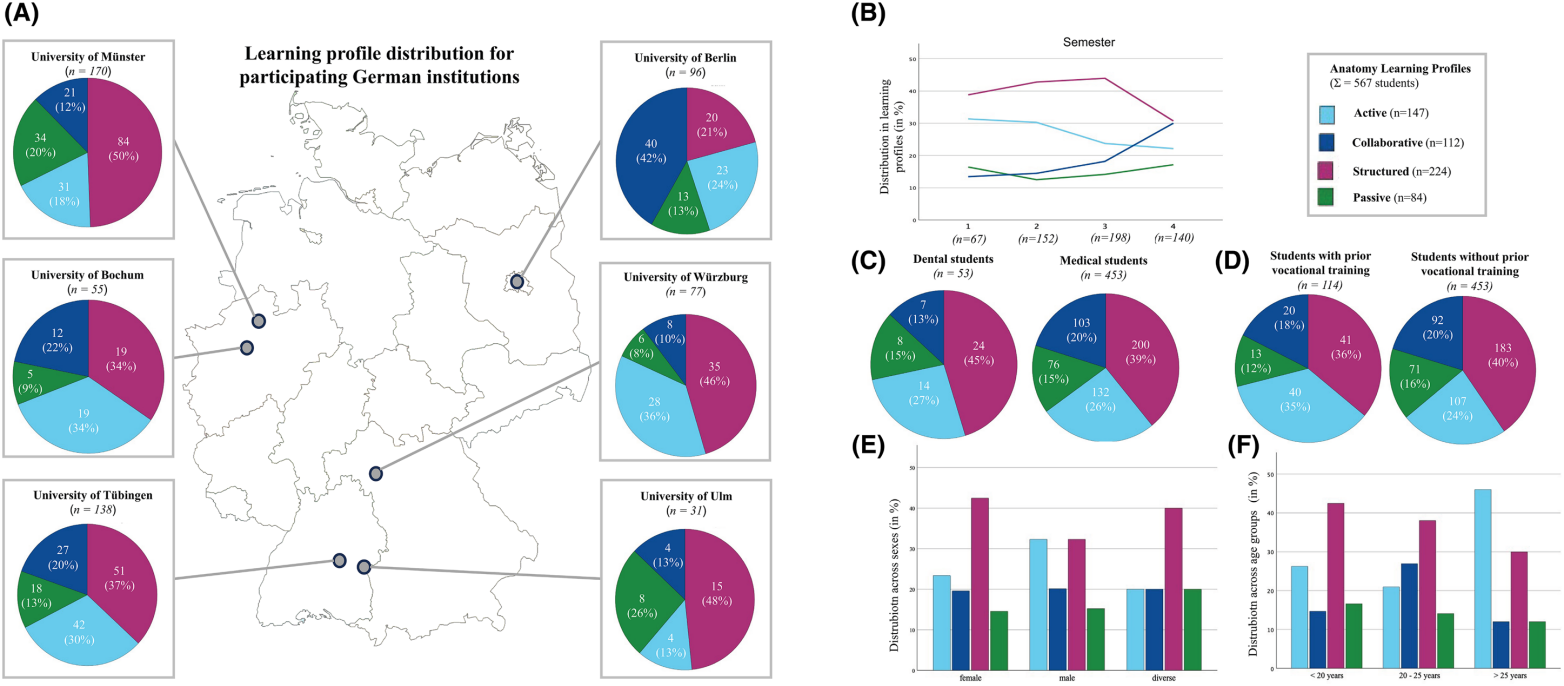
Characterization of the four Anatomy Learning Profiles (ALPs). (A) Map shows ALP distribution across the six participating German medical institutions. Pie charts for each faculty show the proportion of different ALPs among the study participants. (B) Line graph shows a semester‐wise comparison of the ALPs. (C) Pie charts compare the ALP distribution between dental and medical students. (D) Pie charts compare ALP distribution between students with and without prior vocational training. (E and F) Bar graphs show the standardized frequencies of gender and age. The color coding across all panels is as follows: Light blue (Active ALP), dark blue (Collaborative ALP), burgundy (Structured ALP), and green (Passive ALP).

Analysis of profile distributions across the six German medical institutions revealed notable institutional differences (Figure 2A). A clear distinction emerged between Berlin, the single site implementing a modular curriculum (i.e., anatomy integrated into a clinical curriculum), and the remaining sites following traditional subject‐based approaches. The Structured ALP emerged as the predominant profile across institutions (*n* = 224), though its prevalence varied markedly, peaking at 50% in Münster (*n* = 84) and reaching its lowest point in Berlin at 21% (*n* = 20).

Berlin's unique profile distribution is further characterized by the highest proportion of Collaborative ALP among all institutions (42%, *n* = 40), contrasting with the Structured ALP predominance elsewhere. Similar patterns emerged between the students of Ulm and Würzburg, both showing high proportions of Structured ALP (48%, *n* = 15 and 46%, *n* = 35, respectively); though, the students of Ulm displayed a distinctive elevation in Passive ALP representation (26%, *n* = 8). The Active ALP, ranking second in overall frequency (*n* = 147), showed prominence within Bochum's and Würzburg's student populations, comprising 34% (*n* = 19) and 36% (*n* = 28) of their respective student populations.

#### Temporal evolution

Distinct trends also emerged in temporal evolution across semesters (Figure 2B). The Structured ALP was more common through the first three semesters before decreasing in semester 4. The Collaborative ALP increased, particularly in semester 4, and Active ALP demonstrated a gradual decline over time. The Passive ALP varied across semesters. To further explore these trends, the underlying student distribution was examined. Of the total 567 students, the cohort sizes varied significantly across semesters: 1st semester (67 students, 11.8%), 2nd semester (152 students, 26.8%), 3rd semester (198 students, 34.9%), and 4th semester (140 students, 24.7%). The Structured ALP participation peaked at 87 students in the third semester, then decreased to 43 students in the fourth semester. The Collaborative ALP participation gradually increased from 9 students in the first semester to 42 students in the fourth semester. The Active ALP participation declined from 47 students in the third semester to 31 students in the fourth semester. The Passive ALP participation ranged from 11 to 28 students across all semesters.

#### Study program

Study program comparisons indicated some differences between dental and medical students (Figure 2C). Dental students (*n* = 53) typically demonstrated Structured (45%) and Active (27%) approaches, with lower frequencies of Passive (15%) and Collaborative (13%) ALPs. Medical students (*n* = 453) showed a more even distribution across profiles, with Structured (39%), Active (26%), Collaborative (20%), and Passive (15%) ALPs.

#### Prior vocational training

Prior vocational training appears to relate to ALPs (Figure 2D). Students with prior training (e.g., in nursing) showed slightly higher proportions of both Structured (36%) and Active (35%) ALPs compared to those without training.

#### Demographic factors

Demographic patterns suggest some variations in both gender and age distributions (Figure 2E,F). Female students showed a higher proportion of structured ALPs, while male students displayed a more even distribution between structured and active ALPs. Age was also related to ALPs, whereby students under 20 years of age were more frequently allocated to the structured ALP, those between 20–25 years of age displayed a more mixed distribution, and students over 25 years of age showed an increased representation in active ALPs.

### Academic success of the anatomy learning profiles (RQ3)

**FIGURE 3:**
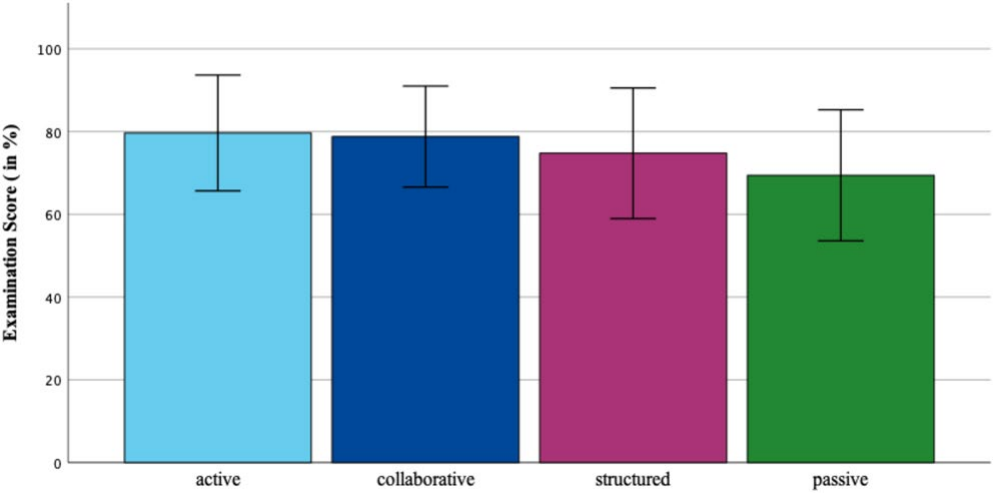
Comparison of academic success in anatomy (i.e., the normalized examination results) across the four ALPs. Structured and Active ALPs achieved higher mean scores compared to Passive and Collaborative ALPs. Bars represent mean values, with error bars indicating Standard Deviation (*σ*).

Analysis of academic performance was based on objective anatomy examination scores that students entered into the questionnaire, normalized to a 0–100% scale. One‐way ANOVA revealed significant differences in examination performance across profiles, F(3, 280) = 5.31, *p* < 0.001 (Figure 3). The active ALP (*Mean* = 79.66, *σ* = 13.99) and collaborative ALP (*M* = 78.78, *σ* = 12.20) significantly outperformed the passive ALP (*M* = 69.42, *σ* = 15.85; *p* = 0.002, and *p* = 0.009, respectively).

### Motivational patterns across ALPs (RQ4)

**FIGURE 4:**
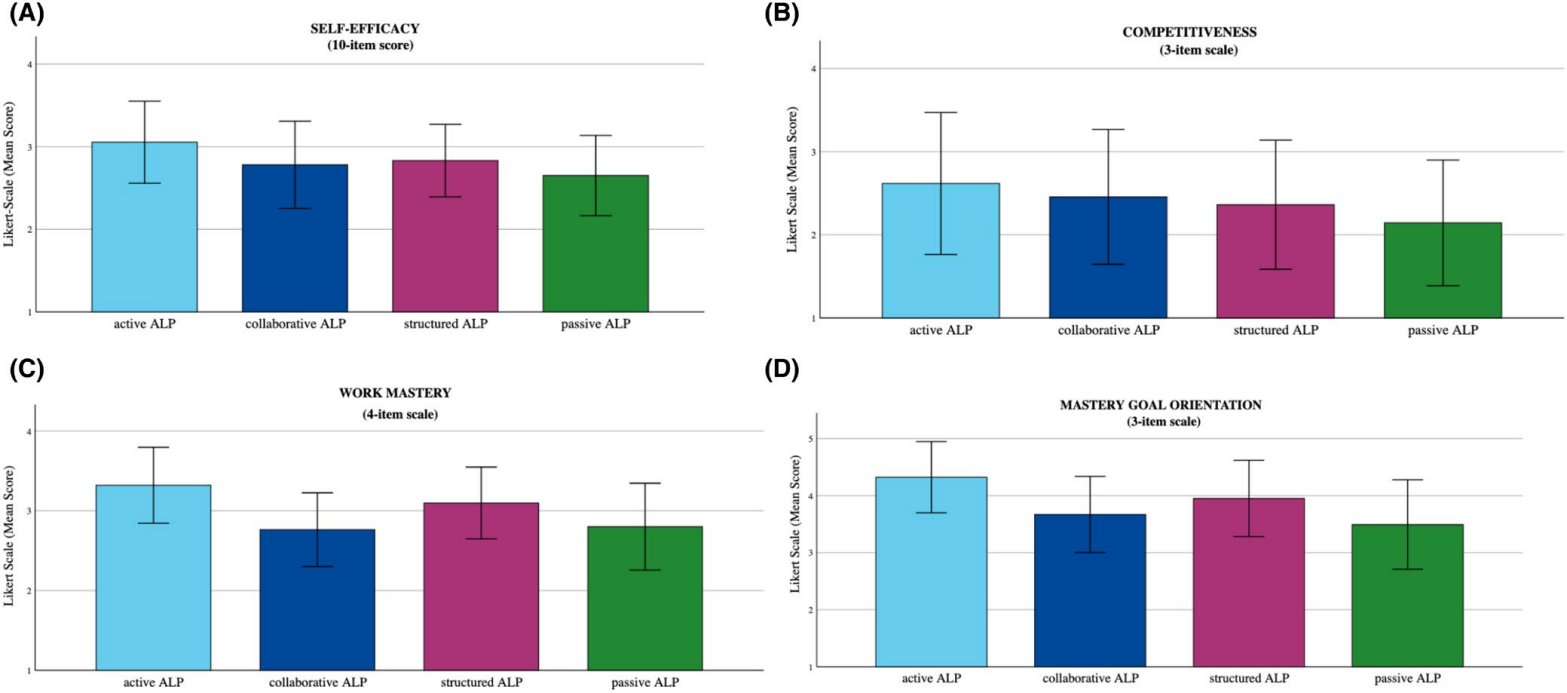
Relationships between ALPs and four dimensions of academic motivation. Analysis of Self‐Efficacy (A), Competitiveness (B), Work Mastery (C), and Mastery Goal Orientation (D). Self‐Efficacy, Competitiveness, and Mastery Goal Orientation were measured on a five‐point Likert scale, while Work Mastery utilized a four‐point Likert scale. Bars represent mean values for each ALP category, with error bars indicating standard deviation (*σ*).

One‐way ANOVA revealed significant differences across all four motivational dimensions (Figure 4).

The Active ALP consistently demonstrated higher scores across all motivational dimensions. Students within this profile showed the highest levels of self‐efficacy, competitiveness, work mastery, and mastery goal orientation. Work mastery and mastery goal orientation emerged as particularly useful differentiators between the ALPs, with notably large statistical effect sizes (F(3,563) = 33.95, partial *η* ^2^ = 0.168 and F(3,563) = 37.98, partial *η* ^2^ = 0.153 respectively, both *p*s < 0.001). A consistent hierarchy emerged in the results whereby Active ALP students had the highest motivation scores, followed by Structured ALP students. Passive and Collaborative ALP students generally showed lower motivation scores.

## DISCUSSION

Using a person‐centered approach, this multicenter study investigated Anatomy Learning Profiles (ALPs) among medical and dental students across six German institutions. By examining how students combine multiple learning strategies, this research moved beyond traditional variable‐centered approaches to address critical gaps in learning strategies research.

### Students' learning behaviors were represented by four anatomy learning profiles

Latent Profile Analysis identified four distinct ALPs that largely aligned with profiles previously described by Otto et al.,^8^ though with notable nuances. The earlier study found Collaborative ALP students characterized by higher frequencies of metacognitive and resource‐related learning strategies, while the current study revealed closer similarities of this specific profile with Passive ALP students. The similarity of the core profiles across multiple German medical institutions, including both medical and dental students studying various anatomical subdisciplines, suggests potential consistency in learning strategy approaches within this educational context; though broader generalizability requires further cross‐cultural, cross‐professional, and longitudinal analysis. This robust ALP classification system could serve as a foundation for investigating the relationship between learning strategies and academic outcomes, potentially offering greater predictive value than examining individual learning strategies that may not fully capture the multifaceted nature of anatomy learning behaviors.

The stability of profiles across different studies (e.g., Otto et al.,^8^) has further implications for anatomy education. Firstly, it suggests that these learning patterns may be rooted in deeper individual differences or preferences rather than being purely situational responses (i.e., context‐dependent). This insight could guide the development of more personalized teaching and learning approaches that acknowledge and accommodate these fundamental learning patterns. For instance, an AI system could assess a student's learning profile through early interaction patterns and subsequently tailor content delivery, practice exercises, and feedback mechanisms to align with their profile‐specific strengths and preferences. Secondly, the consistency of findings between medical and dental students in our sample suggests that these learning patterns may be applicable across closely related health professions within German educational contexts. Further research with more diverse groups of healthcare professionals across different cultural settings is needed to determine whether these profiles reflect truly universal approaches to learning in medical education and whether they are generalizable to subject matter other than anatomy.

Another result points to institutional differences in the distribution of the respective ALPs. While most institutions with non‐modularized curricula showed a predominance of Structured ALPs, modularized curricula fostered a noticeably higher proportion of Collaborative ALPs. This supports the notion that modular curricula may enhance student collaboration and deepen approaches to learning.^11^, ^12^ The modular curricular format creates conditions more conducive to peer interaction, group problem‐solving, and collaborative knowledge construction.

The semester‐wise comparison suggests that ALPs are not fixed traits but rather represent dynamic adaptations as students' progress through medical school. The rise in Collaborative ALP membership in later semesters might reflect the increasing complexity of advanced topics, which benefit from elaboration, critical thinking, and peer‐based learning rather than terminology‐based sessions typical in the first semester. Similarly, the decline in Structured ALP membership may indicate growing student confidence and autonomy in learning. This pattern aligns with theoretical perspectives on learning development that suggest students often progress from more structured, instructor‐dependent approaches toward more self‐directed and collaborative strategies as they gain expertise.^35^ These shifts likely represent a natural maturation in students' learning behaviors. Through exposure to various teaching methods (e.g., Bostedt et al.,^36^; Darici et al.^37^, ^38^, ^39^) and continuous self‐reflection, students may develop more sophisticated cognitive skills, enabling them to use their learning time more efficiently. This “learning to learn” phenomenon may partly explain students' gradual transition from heavily structured approaches to more flexible and collaborative learning strategies. The temporal dimension of profile development has important implications for educational interventions. It suggests that different support strategies may be optimal at different stages of anatomical sciences education. Rather than attempting to shift students toward a single “ideal” profile, educators might focus on helping students develop versatility in their learning approaches and supporting strategic shifts in strategy usage as curricular demands evolve.

In contrast to Otto et al.,^8^ gender differences were pronounced in the Structured ALP, with female students showing a higher representation. Age also influenced ALP distribution, with older students gravitating toward Active ALP membership, possibly due to greater maturity and prior educational experiences.

Contrary to previous studies,^40^ dental students demonstrated a sophisticated use of learning strategies. The preference for Structured and Active ALP membership challenges stereotypes about dental students' reliance on practical rather than intellectual learning strategies. These findings also align with recent studies indicating dental students' effective adaptation to diverse learning modalities.^41^

Students with prior vocational training showed a higher representation in the Active ALP, suggesting that practical experience may enhance overall engagement and effort. This finding aligns with research highlighting the positive impact of vocational training on the integration of theoretical knowledge with practical application.^42^ However, it is important to note that vocational training in the study sample was strongly confounded with age, as students with prior vocational experience were, on average, significantly older than their counterparts entering directly from secondary education. This age difference could be the more determining factor in explaining the higher engagement levels, as mature students demonstrated Active ALP membership.

### Anatomy learning profiles are associated with academic success and student motivation

Active and Collaborative ALP memberships were associated with superior academic performance, highlighting the complexity of learning strategies in anatomy education. These findings underscore the importance of combining active engagement, critical thinking, and elaboration, rather than relying solely on extensive rehearsal.

Active ALP students consistently demonstrated the highest levels of self‐efficacy, competitiveness, work mastery, and mastery goal orientation. These motivational dimensions provide insights into how self‐efficacy, competitiveness, work mastery, and mastery goal orientation interact with learning strategies. Whether the significant associations between work mastery and mastery goal orientation enhance educational outcomes deserves more focus in future investigations. By examining the interplay of these motivational constructs, researchers and educators could develop more targeted approaches to supporting student learning across different Anatomy Learning Profiles.

### Study limitations and future directions

This study has several limitations. The cross‐sectional design limits causal interpretations regarding the development and stability of ALPs over time. While this work allowed for observations of profile distributions across semesters, it was not designed to track individual students' profile development longitudinally. This makes it challenging to determine whether observed semester differences represent genuine developmental progressions or cohort effects. Additionally, the timing of the study, conducted during regular curriculum delivery, may not fully capture learning strategies used throughout the semester. The exclusive focus on German medical schools may limit the generalizability of these findings to other educational systems within Europe or beyond, highlighting the need for cross‐cultural comparisons in future research.

With a response rate of around 20%, the possibility of selection bias in the sampling must be considered. Even though the survey was conducted anonymously, it is possible that students who felt more confident about their academic standing were more inclined to participate. This potential self‐selection effect suggests the sample may not fully represent the entire student population's academic performance spectrum.

The Cronbach's alpha values for work mastery (0.638) and mastery goal orientation (0.619) scales were below the commonly accepted threshold of 0.70 for good internal consistency. This limitation may impact the reliability of findings related to these specific motivational constructs.

Future research should address these limitations by prioritizing longitudinal study designs with repeated measurements of the same individuals to investigate ALP stability and profile transitions over time. This approach would help determine whether profiles represent relatively stable individual differences or situational adaptations that evolve with educational context and experience. Standardized, anatomy‐specific assessment tools could further improve measurement consistency and comparability across studies, while cross‐validation of findings across anatomical subdisciplines and the inclusion of clinical disciplines would further strengthen the theoretical framework. Given the relatively small sub‐sample size of dental students, future studies could focus on this subgroup to develop more nuanced profile analyses. Additionally, intervention studies could examine whether targeted educational coaching can facilitate beneficial profile transitions at different educational stages, helping students develop more adaptable and effective approaches to anatomy learning as they progress through their medical training. Future research may benefit from incorporating practical performance data and exploring strategies to encourage broader participation across all achievement levels.

Studying the effectiveness of educational tools in supporting different ALPs and exploring how shifts in ALPs influence academic success are topics ripe for investigation. Implementing these findings could involve developing “profile‐aligned teaching methods”, resources, and inclusive learning environments that promote individualized learning based on ALPs. AI systems could play a crucial role here by analyzing student learning patterns and automatically adjusting content delivery, pacing, and presentation formats to match individual profiles, creating truly adaptive educational experiences. At the student level, understanding individual ALPs could empower learners to make informed decisions about learning strategies and resource utilization, transforming their engagement with educational content.

## CONCLUSION

This study identifies four distinct Anatomy Learning Profiles (Active, Collaborative, Structured, and Passive) that reflect how medical and dental students combine multiple learning strategies to study anatomy. These results advance the emerging person‐centered paradigm in anatomy education research and provide empirical support for developing targeted educational interventions that align with students' learning behavior, laying the groundwork for evidence‐based curriculum design in anatomy education.

## AUTHOR CONTRIBUTIONS

**Birte Barbian:** Conceptualization; methodology; software; investigation; formal analysis; visualization; project administration; writing – original draft; writing – review and editing; data curation. **Thomas Shiozawa:** Data curation; writing – original draft; writing – review and editing. **Morris Gellisch:** Data curation; writing – original draft; writing – review and editing. **Irene Brunk:** Data curation; writing – original draft; writing – review and editing. **Katharina Langer‐Fischer:** Data curation; writing – original draft; writing – review and editing. **Nicole Wagner:** Data curation; writing – original draft; writing – review and editing. **Sina Benker:** Writing – original draft; writing – review and editing. **Michelle Bellstedt:** Writing – original draft; writing – review and editing. **Nils Otto:** Data curation; writing – original draft; writing – review and editing; validation. **Dogus Darici:** Conceptualization; methodology; software; data curation; investigation; validation; formal analysis; supervision; resources; project administration; visualization; writing – original draft; writing – review and editing.

## FUNDING INFORMATION

This project received no specific funding.

## CONFLICT OF INTEREST STATEMENT

There are no conflicts of interest to declare.

## Supporting information


**Figure S1:** Different Latent Profile solutions (2‐profile, 3‐profile, 4‐profile, and 5‐profile solutions) are shown. The decision on the final four‐profile model was based on statistical fit indices (see Table S1) and on theoretical arguments.

**Data S1:** Supplementary Information.

**Table S1:** Psychometric Properties of Anatomy Examinations Across Six Participating Institutions (Summer Semester 2024).
