# Supplementary figures and images for: Anatomy learning profiles in relation to student motivation and academic success: A multi‐center cross‐sectional study
Source: Anat Sci Educ. 2025 Jun 27;18(10):1057–69. doi: 10.1002/ase.70065 (PMC12511653; doi:10.1002/ase.70065)

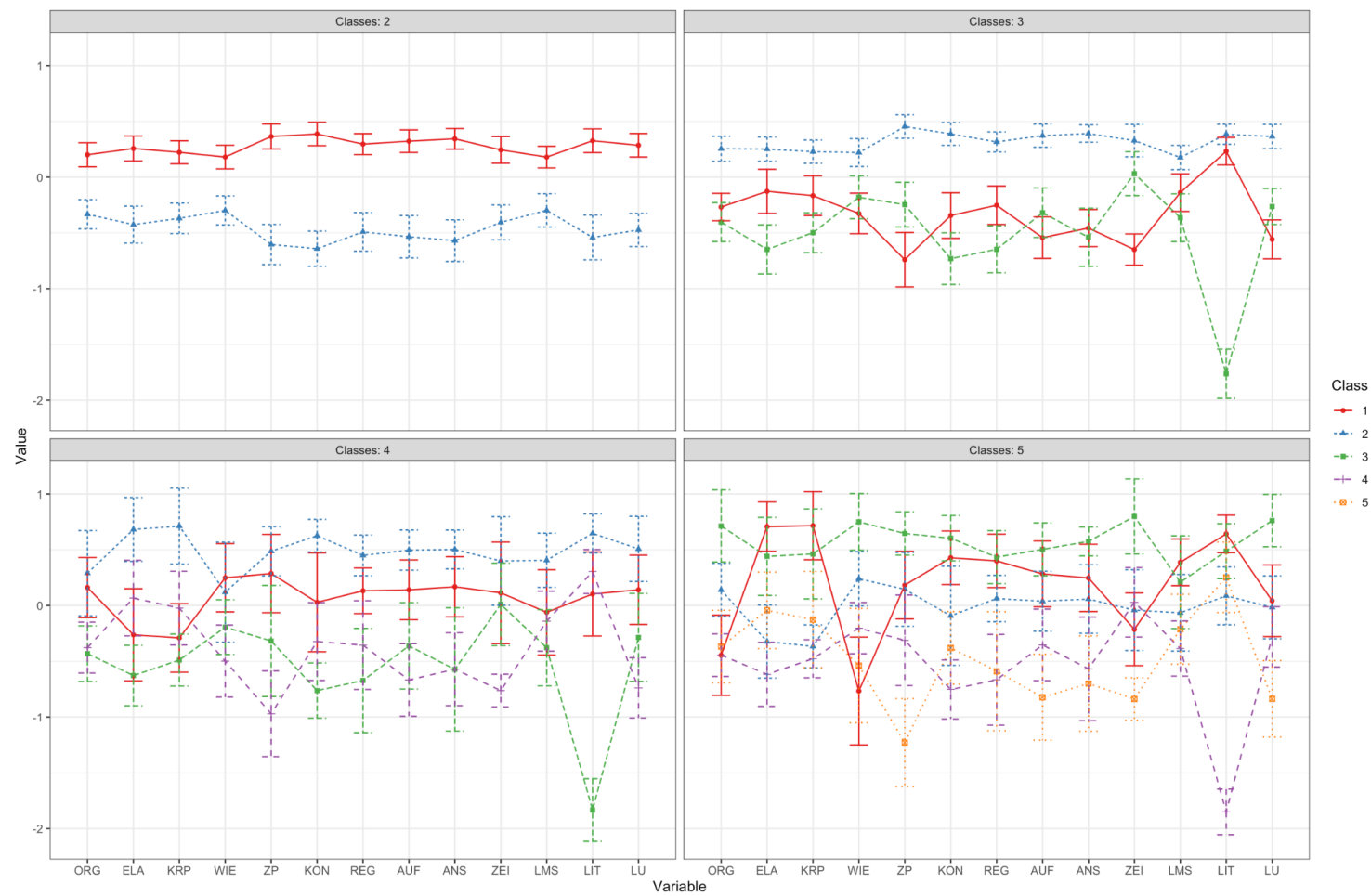

Supplement: Supplementary file 1 — Figure S1: Different Latent Profile solutions (2‐profile, 3‐profile, 4‐profile, and 5‐profile solutions) are shown. The decision on the final four‐profile model was based on statistical fit indices (see Table S1) and on theoretical arguments. [file ASE-18-1057-s001.pdf]
