# Supplementary material for: Anatomy learning profiles in relation to student motivation and academic success: A multi‐center cross‐sectional study
Source: Anat Sci Educ. 2025 Jun 27;18(10):1057–69. doi: 10.1002/ase.70065 (PMC12511653; doi:10.1002/ase.70065)
Supplement: Supplementary file 2 — Data S1: Supplementary Information. [file ASE-18-1057-s003.docx]

**Supplementary Appendix**

**Theoretical framework**

Given that learning strategy research in higher education has a long-standing tradition, a brief introduction to different theoretical frameworks is warranted. There are several strands to conceptualize learning strategies, e.g., self-regulated learning, approaches to learning, and learning pattern research. The current study, similarly to the study of Otto et al. (2024), and Odontides et al. (2024), refers to the patterns of learning research, which will be introduced in the following section. This strand focuses on cognitive, metacognitive, and resource-related behaviors of learning (Vermunt and Donche, 2017).

One instrument within this learning patterns tradition is the LIST-K (Klingsieck et al., 2018), a condensed and modified version of the original Learning Strategies in Studies (LIST) questionnaire in German language. The LIST-K serves as a bridge between theoretical learning pattern models and empirical research. This 39-item questionnaire draws from various theoretical concepts and sources, including Kirby's (1988) learning strategy-concept, the Motivated Strategies for Learning Questionnaire (MSLQ) by Pintrich et al. (1993), and the Learning and Study Strategies Inventory (LASSI) by Weinstein (1988).

The LIST-K incorporates thirteen learning strategies (first-order factors), categorized into four principal domains (second-order factors):

1. Cognitive strategies: organization, elaboration, critical thinking, and rehearsal

2. Metacognitive strategies: planning, monitoring, and regulating

3. Management of internal resources: attention, effort, and time management

4. Management of external resources: peer learning, literature, and study environment

These 13 goal-oriented learning behaviors may be well-suited for understanding anatomy education because they address the challenges students face in mastering anatomical sciences. The cognitive strategies support the integration of extensive anatomical terminology and three-dimensional spatial relationships that define anatomical structures. Metacognitive strategies may be essential for navigating the voluminous content within limited curricular time—a particular challenge in modern anatomy curricula. The resource management strategies might be crucial for anatomy learning, where students must effectively utilize diverse learning materials including cadaveric specimens, histological slides, anatomical models, and cross-sectional images.

While learning strategies represent behavioral patterns, they are driven by underlying motivational mechanisms (Hayat et al., 2020; Reynolds et al., 2024). Self-efficacy, competitiveness, work mastery, and mastery goal orientation were chosen for their potential to illuminate the underlying motivational mechanisms that drive students‘ learning strategy use. Each construct represents a different dimension of student motivation that has shown implications for academic performance:

*Self-efficacy.* Self-efficacy, a concept introduced by Bandura, refers to an individual's belief in their capacity to perform specific tasks successfully (Reynolds et al 2024). This psychological construct has emerged as a key factor in medical education with wide ranging positive impacts (Costa Filho et al., 2022). Students and faculty with higher self-efficacy show better emotional wellbeing, less burnout, and improved stress management. Stankov and Lee (2014) have found that self-efficacy is one of the strongest non-cognitive predictors of academic success. It enhances academic performance through better learning strategies, persistence, and study approaches, as demonstrated in anatomical education where self-efficacy relates to a student's confidence in mastering tasks within the anatomy curriculum, such as dissection, understanding anatomical concepts, and applying this knowledge to clinical scenarios (Burgoon et al, 2012; Selvig et al 2015).

*Competitiveness.* Medical students routinely encounter high pressure competitive environments in their academic pursuits. Competitiveness has been consistently identified as an intrinsic trait of medical education (Lempp and Seale, 2004; Humayon and Shoaib, 2019). The competitiveness questionnaire assesses an individual's orientation towards competition through items measuring enjoyment of competitive situations, desire to outperform others, and increased effort in competitive contexts. These items collectively evaluate a person's tendency to engage in and positively respond to competitive environments, reflecting their motivation for social comparison and performance enhancement under competitive pressure.

*Work mastery.* The concept of mastery has evolved significantly in academic literature, from discussions of mastery orientation and achievement goal theory (Nicholls, 1984; Elliot and McGregor 2001; Dweck, 2004) to phenomenological understandings of embodied practice (Figueiredo & Ipiranga, 2015). Mastery represents a transformative state where knowledge becomes integrated through bodily engagement, requiring mechanical skill, judgment, and dexterity that shape both professional and personal identities (Darici et al., 2022). This challenges traditional views separating professional knowledge from bodily experience, suggesting mastery as a holistic state where knowledge, practice, and identity intertwine through embodied learning.

*Mastery goal orientation.* Mastery goal orientation, a construct in achievement motivation theories, emphasizes metacognitive development and learner autonomy over comparative performance metrics (Elliot and McGregor 2001; Payne et al. 2007). In contrast to traditional performance-centered paradigms (e.g., Bellstedt et al., 2024; Brügge et al., 2024; Darici et al., 2024) mastery orientation cultivates sustainable LS and resilient professional development practices, establishing a comprehensive framework for practitioners capable of adapting to evolving disciplinary knowledge throughout their careers (Ross et al., 2021).

Based on these 13 first-order learning strategies Otto et al. (2024) applied a person-centered approach (i.e., a *latent profile analysis*). In contrast to data-driven k-means clustering or exploratory factor analysis, latent profile analysis relies on advanced mathematical algorithms to cluster acquired data and verify profile allocation by fitting hypothetical models. It is able to reveal distinct patterns of learning strategies, leading to the identification of the four ALPs. Otto et al. (2024) found that each profile is defined by a unique combination of cognitive, metacognitive, and resource management strategies:

- *Active ALP students* demonstrate high utilization across all learning strategies. These students effectively integrate critical thinking and elaborating (deep approaches) and rehearsal (surface approaches), while making comprehensive use of recommended and supplementary resources.
- *Collaborative ALP* students show selective strategy implementation, particularly excelling in peer learning, as well as critical thinking, monitoring, and literature utilization. These students show noticeable lower engagement with planning and time-management strategies.
- *Structured ALP* students adopt a more systematic approach, characterized by moderate overall strategy use, with particular strengths in planning and time management. However, these students tend to underutilize cognitive strategies, peer learning, and literature resources.
- *Passive ALP* students display minimal strategy utilization across all categories, primarily relying on peer learning and simplified literature. Their learning media use suggests reliance on repetition-based learning.

Furthermore, Otto et al. (2024) found that Passive ALP students were slightly older. Gender distribution was generally uniform, with two notable exceptions: A higher proportion of female students within the Active ALP, as well as within the Passive ALP, suggesting a potential bimodal distribution among female learning behavior.

While this study identified distinct Anatomy Learning Profiles, these profiles might not represent fixed or unchangeable approaches to learning. Rather, they may capture snapshots of strategy combinations that students employ at particular points in their educational journey. Educational research suggests that learning approaches evolve as students’ progress through different educational levels, respond to varying curricular demands, and develop metacognitive awareness through educational coaching and experience (Vermunt & Donche, 2017). This temporal dimension of learning strategy development is a critical aspect of the conceptual framework and distinguishes the learning profiles approach from more static conceptions of learning styles.

**Supplementary Reference List**

Bellstedt, M., Holtrup, A., Otto, N., Berndt, M., Scherff, A. D., Papan, C., Robitzsch, A., Missler, M., & Darici, D. (2024). Gaze cueing improves pattern recognition of histology learners. *Anatomical Sciences Education, 17*(7):1461-1472. <https://doi.org/10.1002/ase.2498>

Burgoon, J. M., Meece, J. L., & Granger, N. A. (2012). Self-efficacy's influence on student academic achievement in the medical anatomy curriculum. *Anatomical Sciences Education, 5*(4), 249–255.<https://doi.org/10.1002/ase.1283>

Brügge, E., Ricchizzi S., Arenbeck, M., Keller, M. N., Schur, L., Stummer, W., Holling, M., Lu, M. H., & Darici, D. Large language models improve clinical decision making of medical students through patient simulation and structured feedback: a randomized controlled trial.

*BMC Medical Education, 24*(1):1391. <https://doi.org/10.1186/s12909-024-06399-7>

Darici, D., Missler, M., Schober, A., Masthoff, M., Schnittler, H., & Schmitz, M. (2022). “Fun slipping into the doctor’s role”–The relationship between sonoanatomy teaching and professional identity formation before and during the Covid-19 pandemic. *Anatomical Sciences Education*, *15*(3), 447-463. <https://doi.org/10.1002/ase.2178>

Darici, D., Flägel, K., Sternecker, K., & Missler, M. (2024). Transfer of learning histology: insights from a longitudinal study. *Anatomical Sciences Education, 16*(4):720-732. <https://doi.org/10.1002/ase.2363>

Dweck, C. S., Mangels, J. A., & Good, C. (2004). Motivational effects on attention, cognition, and performance. In D. Y. Dai & R. J. Sternberg (Eds.), *Motivation, emotion, and cognition: Integrative perspectives on intellectual functioning and development* (pp. 41–55). Mahwah, NJ: Lawrence Erlbaum Associates Publishers.

Figueiredo, M. D., & Ipiranga, A. S. R. (2015). How can we define mastery? Reflections on learning, embodiment, and professional identity. *BAR - Brazilian Administration Review, 12*(4), 348–364. <https://doi.org/10.1590/1807-7692bar2015150076>

Nicholls, J. G. (1984). Achievement motivation: Conceptions of ability, subjective experience, task choice, and performance. *Psychological Review, 91*(3), 328–346. <https://doi.org/10.1037/0033-295X.91.3.328>

Payne, S. C., Youngcourt, S. S., & Beaubien, J. M. (2007). A meta-analytic examination of the goal orientation nomological net. *Journal of Applied Psychology, 92*(1), 128–150. <https://doi.org/10.1037/0021-9010.92.1.128>

Selvig, D., Holaday, L. W., Purkiss, J., & Hortsch, M. (2015). Correlating students' educational background, study habits, and resource usage with learning success in medical histology. *Anatomical Sciences Education, 8*(1), 1–11. <https://doi.org/10.1002/ase.1449>

Stankov, L., & Lee, J. (2014). Quest for the best non-cognitive predictor of academic achievement. *Educational Psychology Review, 34*(1), 1–8. <https://psycnet.apa.org/doi/10.1080/01443410.2013.858908>
