# Supplementary material for: Anatomy learning profiles in relation to student motivation and academic success: A multi‐center cross‐sectional study
Source: Anat Sci Educ. 2025 Jun 27;18(10):1057–69. doi: 10.1002/ase.70065 (PMC12511653; doi:10.1002/ase.70065)
Supplement: Supplementary file 3 — Table S1: Psychometric Properties of Anatomy Examinations Across Six Participating Institutions (Summer Semester 2024). [file ASE-18-1057-s002.docx]

| **Supplementary Table 1** | | | |  |
| --- | --- | --- | --- | --- |
| *Psychometric Properties of Anatomy Examinations Across Six Participating Institutions*  *(Summer Semester 2024)* | | | |  |
|  | Reliability  (Cronbach’s Alpha) | Average  Difficulty | Average  Item Dicrimination |  |
| Berlin (*n=96*) | N/A * | 0.77 | N/A * |  |
| Bochum (*n=55*) | N/A** | 0.79 | N/A** |  |
| Münster (*n=170*) | 0.92 | 0.69 | 0.31 |  |
| Tübingen (*n=138*) | 0.68 | 0.56 | 0.27 |  |
| Ulm (*n=31*) | 0.68 | 0.45 | 0.21 |  |
| Würzburg (*n=77*) | 0.81 | 0.74 | 0.38 |  |
| Note. Average item discrimination refers to the mean item-total discrimination index across all items.  * Data could not be included due to formal restrictions on data availability.  ** Only pass/fail outcomes were available; no item-level scores or test metrics were computed. | | | |  |
